# Fetuin-A protein distribution in mature inflamed and ischemic brain tissue

**DOI:** 10.1371/journal.pone.0206597

**Published:** 2018-11-09

**Authors:** Miriam Christina Heinen, Anne Babler, Joachim Weis, Johannes Elsas, Kay Nolte, Markus Kipp, Willi Jahnen-Dechent, Martin Häusler

**Affiliations:** 1 Department of Pediatrics, RWTH Aachen University Hospital, Aachen, Germany; 2 Helmholtz Institute for Biomedical Engineering, Biointerface Laboratory, RWTH Aachen University Hospital, Aachen, Germany; 3 Institute of Neuropathology, RWTH Aachen University Hospital, Aachen, Germany; 4 Institute of Neuropathology, JARA-BRAIN, Jülich, Germany; 5 Institute of Neuroanatomy, RWTH Aachen University Hospital, Aachen, Germany; Texas Tech University, UNITED STATES

## Abstract

**Background:**

The liver-derived plasma protein fetuin-A is strongly expressed during fetal life, hence its name. Fetuin-A protein is normally present in most fetal organs and tissues, including brain tissue. Fetuin-A was neuroprotective in animal models of cerebral ischemia and lethal chronic inflammation, suggesting a role beyond the neonatal period. Little is known, however, on the presence of fetuin-A in mature human brain tissue under different physiological and pathological conditions.

**Methods:**

We studied by immunohistochemistry (IHC) the distribution of fetuin-A protein in mature human brain autopsy tissues from patients without neurological disease, patients with inflammatory brain disorders, and patients with ischemic brain lesions. To identify fetuin-A-positive cells in these tissues we co-localized fetuin-A with GFAP (astrocytes) and CD68 (macrophages, activated microglia).

**Results and discussion:**

Unlike previous reports, we detected fetuin-A protein also in mature human brain as would be expected from an abundant plasma protein also present in cerebrospinal fluid. Fetuin-A immunoreactivity was increased in ischemic white matter and decreased in inflamed cerebellar tissue. Fetuin-A immunostaining was predominantly associated with neurons and astrocytes. Unlike the developing brain, the adult brain lacked fetuin-A immunostaining in CD68-positive microglia. Our findings suggest a role for fetuin-A in tissue remodeling of neonatal brain, which becomes obsolete in the adult brain, but is re-activated in damaged brain tissue. To further assess the role of fetuin-A in the mature brain, animal models involving ischemia and inflammation need to be studied.

## Introduction

Fetuin-A is a member of the cystatin superfamily. It was first described by Pederson in 1944 as a major serum globulin in fetal calves [1]. The human homologue protein in serum was first described by Heremans [2], Schmidt and Bürgi [3]. Later it was characterized as α2 globulin [4] and named α2-Heremans-Schmidt-glycoprotein (AHSG) in honor of the first discoverers. Today, both names are synonymous for this human plasma glycoprotein.

Fetuin-A is a negative acute phase protein [5] with protective effects in inflammation [6, 7] and cerebral ischemia [8]. Multiple other biological functions of fetuin-A have been published. First and foremost fetuin-A has a role in calcified matrix metabolism [9] especially in the prevention of ectopic calcification [10, 11]. Due to its high affinity to hydroxyapatite, fetuin-A regulates mineralized cartilage and bone metabolism. Fetuin-A deficient mice develop post-weaning epiphysiolysis causing distal femur dysplasia and foreshortened hindlimbs [12]. Adult bone turnover and fine structure seem largely unaffected by fetuin-A deficiency [13]. During embryogenesis fetuin-A is expressed in liver, brain, kidney, muscle and bone tissue [14], and is also abundant in plasma and cerebrospinal fluid (CSF) [15]. Thereafter fetuin-A is constitutively produced in the liver and secreted into the blood stream. In humans, the highest serum levels are measured during the 24^th^-30^th^ week of pregnancy. From the 37^th^ week of pregnancy onward, serum levels are stable until adolescence [16].

### Distribution in the brain

Little is known about the role of fetuin-A in the adult human brain. Fetuin-A protein was reported absent in the brain of adult sheep and rats [17–20]. The distribution of fetuin-A in the developing brain was examined in various species. In the developing neocortex fetuin-A co-localized with a population of cells that migrated from the ventricular zone to form the primordial plexiform layer and the early cortical plate [17, 20–23]. Dziegielewska and colleagues [22] observed that the distribution of fetuin-A in the early cortical plate of human embryos of 7 weeks and sheep embryos of 35–36 days was very similar. In human fetuses of 33 weeks of gestational age, fetuin-A staining was negative. Previously, we examined the distribution of fetuin-A in the brain of human and rat fetuses and in newborns [23]. In human tissue, fetuin-A staining decreased with age, but still was found in the cortex of term born neonates. Throughout all gestational ages, fetuin-A immunoreactivity was consistently detected in ependymal cells, the periventricular stem cell layer, the hippocampus, the cerebral cortex, subplate and white matter. Occasionally fetuin-A was detected in the subependymal gap, in basal nuclei and in neurons of the thalamus. In the rat brain, fetuin-A containing cells showed a similar distribution like human tissue. Fetuin-A positive cells were identified as immature neurons in the early neocortex, as mature neurons in the developed hippocampus, as activated microglia in cingular gyrus and periventricular stem cell layer and as astrocytes in the cingular white matter. Fetuin-A staining was detectable until P20 [20], and P28 [23], respectively in two separate studies in postnatal rats.

### Functions of fetuin-A in inflammation and cerebral ischemia

Fetuin-A is a negative acute phase protein whose synthesis is down regulated by the inflammatory cytokines interleukin-1, interleukin-6, tumor necrosis factor alpha (TNFα) [24] and interferon-ɣ [7]. Fetuin-A in turn may counter-regulate inflammation. By binding to spermine it indirectly inhibits the production of TNFα [25], significantly attenuating inflammation in e.g. carrageenan-induced paw edema [6]. Fetuin-A also protected against lipopolysaccharide LPS toxicity in mice [7] and rats [26]. Survival rates of fetuin-A deficient mice with lethal chronic inflammation were significantly lower than those of wild type mice [7]. External supplementation of fetuin-A improved survival while disruption of fetuin-A supplementation led to increased serum levels of high mobility group box 1 nuclear protein (HMGB1) a strong mediator of inflammation of the late immune response, which is released predominantly by macrophages, monocytes and necrotic cells.

Similar mechanisms like in inflammation could be responsible for a neuroprotective effect of fetuin-A in cerebral ischemia. In an animal model of focal cerebral ischemia Wang and colleagues [8] observed a marked increase in fetuin-A serum levels 24h after occlusion of the medial cerebral artery (MCA), which decreased significantly after 72h. Intravenous administration of fetuin-A 15 min after MCA occlusion led to 90% reduction of infarct volume and also attenuated HMGB1 depletion, activation of microglia and macrophages, and of TNF production. According to studies with intravenously administered fluorescein isothiocyanate (FITC) labeled fetuin-A, exogenous fetuin-A protein gained entry across the blood brain barrier into the ischemic brain tissue.

In autoimmune inflammation fetuin-A was associated with disease severity [27] in that fetuin-A CSF levels were significantly increased in patients with active multiple sclerosis and diminished with successful therapy. In autopsy tissue of MS patients, demyelinated lesions in grey and white matter and cerebellar Purkinje cells stained positive for fetuin-A, whereas autopsy tissue from non-neurological patients stained negative [27]. Accordingly, in an animal model of experimental autoimmune encephalitis (EAE), fetuin-A expression co-localized with demyelinated lesions of the spinal cord. Fetuin-A deficient mice demonstrated delayed onset and reduced severity of EAE symptoms [27]. In a subsequent study [28], mice lacking fetuin-A showed reduced lymphocyte and macrophage infiltration into the spinal cord.

### Aim of the study

Here we studied the distribution of fetuin-A during cerebral inflammation and ischemia in humans. We asked whether inflammation or ischemia led to an altered expression of fetuin-A in human brain tissue. Therefore, we studied by immunohistochemistry the distribution of fetuin-A in autopsy tissue of subjects who had suffered from cerebral inflammation or focal ischemia, and of subjects without neuropathological abnormalities. To identify fetuin-A positive cells as astrocytes or activated microglia we performed double immunostaining.

## Methods

### Tissue samples

Human brain tissue was obtained from autopsies carried out between 1999 and 2014 at the Institute of Neuropathology, RWTH University Hospital Aachen. The tissue samples had been acquired from defined standard regions routinely analyzed during neuropathological examination. Tissues were formaldehyde-fixed and paraffin-embedded. This included tissue samples of pallium (cortex and adjacent white matter), basal ganglia, temporal lobe with hippocampus and the surrounding tissue and cerebellum.

Samples used in this study were selected from the tissue bank of the Institute of Neuropathology according to clinical and pathological description, indicating the presence of ischemia, of cerebral infection or of normal brain. Cases were excluded from the ischemic group or the control group when pathology files indicated the presence of an inflammatory or infectious disease. From all samples identified, only those were included for further analysis, that had shown sufficient tissue quality during routine staining. This resulted for instance in the number of 33 cortex/white matter tissue samples from 20 inflammatory cases and 13 samples of the same region from 8 ischemic cases. The study was approved by the Ethics Committee of the Medical Faculty of RWTH Aachen University (EK 90/17). All Tissue samples were processed anonymously.

Control group: Brain autopsy tissue without pathological changes was selected for control. Patients in this group had suffered from non-neurological diseases, such as cardiac decompensation or neoplastic disorders not affecting the brain. 14 control cases of patients, 43 to 79 years old, were included for the examination of pallium (cortex and adjacent white matter), basal ganglia, temporal lobe with hippocampus and the surrounding tissue and cerebellum.

Inflammatory group: Human brain tissue assigned to this group showed inflammatory changes diagnosed by a qualified neuropathologist (JW, KN) Clinical diagnoses included meningitis, encephalitis or septicemia. In total, 20 autopsy cases were examined. Patient age ranged from 23 months to 74 years. Samples from pallium, basal ganglia, temporal lobe and cerebellum were examined.

Ischemic group: Cases were assigned to this group if an ischemic cerebral infarction had been found at autopsy that was also mirrored in clinical diagnosis. Altogether, 14 cases of patients aged 52 to 86 years were selected. The examination was restricted to the ischemic areas, identified by a qualified neuropathologist. For semiquantitative assessment, we only evaluated tissue samples of the cerebral cortex and adjacent white matter, due to small amounts of ischemic tissue samples available from other brain regions.

### Immunohistochemistry

The brain tissue was fixed in a 10% formaldehyde solution for several weeks before dissection of the tissue samples. The tissue samples were kept in a 4% formaldehyde solution and were then embedded in paraffin in an automated tissue processor (TISSUE-TEK VIP 2000, Sakura). Tissue sections of 1 μm were used for all staining procedures. In brief: For deparaffination, the tissue sections were diluted in xylene. Then they were dehydrated in increasing concentrations of ethanol (70–100%). After washing in phosphate buffered saline (PBS), antigen retrieval was performed in a steamer, using citrate buffered saline for chromogenic fetuin-A staining and double immunofluorescence staining with Glial Fibrillary Acid Antigen (GFAP), or Tris-EDTA buffer respectively for double immunofluorescence staining of fetuin-A and CD68. The detailed staining protocols can be viewed at protocol.io (PROTOCOL see next sections).

### Chromogenic fetuin-A staining

In brief: For fetuin-A staining, we used a monoclonal IgG2a mouse-anti-human antibody (clone MAHS-1, dilution 0.1–0.5 μg/mL), raised against purified human fetuin-A in our laboratories. Antibody specificity had been confirmed by Western blotting [29]. Antibodies were diluted in a 1% dilution of Bovine serum albumin (BSA) in phosphate-buffered saline (PBS) and were immediately applied to the re-hydrated sections. Bound antibody was detected using Dako REAL Detection System, which employs APAAP immunochemistry and fast red chromogenic substrate (Dako K5000, Glostrup, Denmark) following the manufacturers protocol. Counterstaining was employed with Mayer´s hematoxylin solution (Roth, T160.1, one minute). The slides were then washed in demineralized water and dehydrated in graded alcohol (concentrations from 70% to 100%). After placing in xylene, the sections were mounted (Roth, T160.1) and covered using coverslips. For details see http://dx.doi.org/10.17504/protocols.io.sydefs6 [PROTOCOL DOI].

### Double immunofluorescence staining of fetuin-A and GFAP

In brief: This staining was performed to detect fetuin-A in astrocytes. Glial Fibrillary Acid Antigen (GFAP) was marked by a polyclonal rabbit-anti-human antibody (Spring Bioscience Cat# E18320, RRID:AB_1661177, dilution 1:50) and a polyclonal goat-anti-rabbit Alexa Fluor 488 conjugated secondary antibody (Thermo Fisher Scientific Cat# A-11070, RRID:AB_2534114, dilution 1:300). Fetuin-A was detected by using the above mentioned monoclonal mouse-anti-human antibody, diluted 1.0 μg/ml. Antibody binding was detected by tyramide signal amplification using a secondary biotinylated polyclonal goat-anti-mouse antibody (Dako Cat# E0433, RRID:AB_2687905, dilution 1:300) and a Tyramide Signal Amplification Kit (Life Technologies, Carlsbad, USA, T-20933). To minimize lipofuscin autofluorescence, sections were counterstained with Sudan Black (Sigma-Aldrich, Munich, Germany, 199664, dilution 0.3% in 70% ethanol, 5 minutes). Nuclei were stained with DAPI (Sigma-Aldrich, Munich, Germany D9542, dilution 0.25 μg/ml, 5 minutes). Sections were mounted with Immumount (Thermo Scientific, Waltham, USA, 9990402) and stored at 8°C in the dark. For details see http://dx.doi.org/10.17504/protocols.io.syeefte [PROTOCOL DOI].

### Double immunofluorescence staining of fetuin-A and CD68

This staining was performed to detect fetuin-A in activated microglia. In brief: CD68 was stained using a monoclonal mouse-anti-human antibody (Dako Cat# M0814, RRID:AB_2314148, clone KP1, dilution 1:50) and a polyclonal goat-anti-mouse Alexa Fluor 488 conjugated secondary antibody (Thermo Fisher Scientific Cat# A-11029, RRID:AB_2534088, dilution 1:300). Fetuin-A was detected as described above. Staining with Sudan Black and DAPI, washing, mounting and storing was performed as above. For details see http://dx.doi.org/10.17504/protocols.io.syfeftn [PROTOCOL DOI].

### Positive and negative controls

Sections of liver tissue served as positive controls for fetuin-A staining in each experiment. Negative controls were carried out during every experiment by incubation with 1% BSA in PBS instead of primary antibody. Immunostainings were only included in this study if positive and negative controls showed the appropriate result. Before double staining of fetuin-A and GFAP, possible cross-reactions between the primary and secondary antibodies of different species were excluded. For this purpose, we applied the Alexa Fluor 488 conjugated anti-rabbit antibody on the primary mouse-anti-human fetuin-A antibody. The secondary biotinylated goat-anti-mouse antibody was applied after tissue incubation with the primary rabbit-anti human GFAP antibody. Both stainings showed a negative result.

### Imaging

Digital photos were recorded using Leica DM6000B and Leica DMRX microscopes, a JVC KY-F75U digital camera and DISKUS imaging software (Technisches Büro Hilgers, Königswinter, Germany, Version 4.80.3479). Digital images were enhanced using Adobe Photoshop CS3 or GNU Image Manipulation Program (GIMP 2.8.16) image processing software.

### Semiquantitative assessment of fetuin-A immunostaining

To maximize spatial information, brain regions were subdivided as follows: neocortex: subpial, perivascular, cortex and adjacent white matter tissue; basal ganglia: perivascular tissue, fiber tracts, neurons; temporal lobe: cortical and white matter, perivascular and periventricular tissue, choroid plexus, dentate gyrus and cornu ammonis, cerebellar tissue: white matter, granular cell layer, Purkinje cells. Fetuin-A immunostaining was scored as 0, no fetuin-A positive cell per high power field (10x lens); 1, one fetuin-A positive cell per view field; 2, 2–5 fetuin-A positive cell per high power field; and score 3, more than five and focal accumulation of fetuin-A positive cell per high power field. Specimens were observer-blinded and evaluated three times by one observer. Mean scores of the subregions were calculated and further analyzed by independent two sample student´s t-test, Mann-Whitney test and Chi-square test or Fisher’s exact test, as appropriate for the respective sample sizes, using Microsoft Excel 2013 and GraphPad Prism (ver 5.0c for Mac OS X) software. Throughout the text we indicate p-values with three digits accuracy. We considered differences statistically significant if p < 0.05.

### Fetuin-A distribution in pathogen-associated inflammation

Neuropathological findings of 11 inflammatory cases were selected, which reported persisting pathogens in brain tissue. Routine H&E as well as selective stain histology of consecutive brain tissue sections were compared with fetuin-A stained sections to co-localize fetuin-A accumulation and pathogens.

### Local staining in ischemic tissue

Ischemic brain areas were identified by H&E histology. The defined ischemic brain areas were evaluated as either positive or negative for fetuin-A. A total of 19 tissue samples from 14 different cases was assessed.

### Double staining

The stained tissue was screened for co-localization in both relevant emission spectra. When co-localization of antigens in a cell was observed, the numbers of cells showing co-localization were counted in 3 neighboring fields imaged with a x 20 lens.

## Results

### Fetuin-A distribution in healthy adult brain

**Fig 1:**
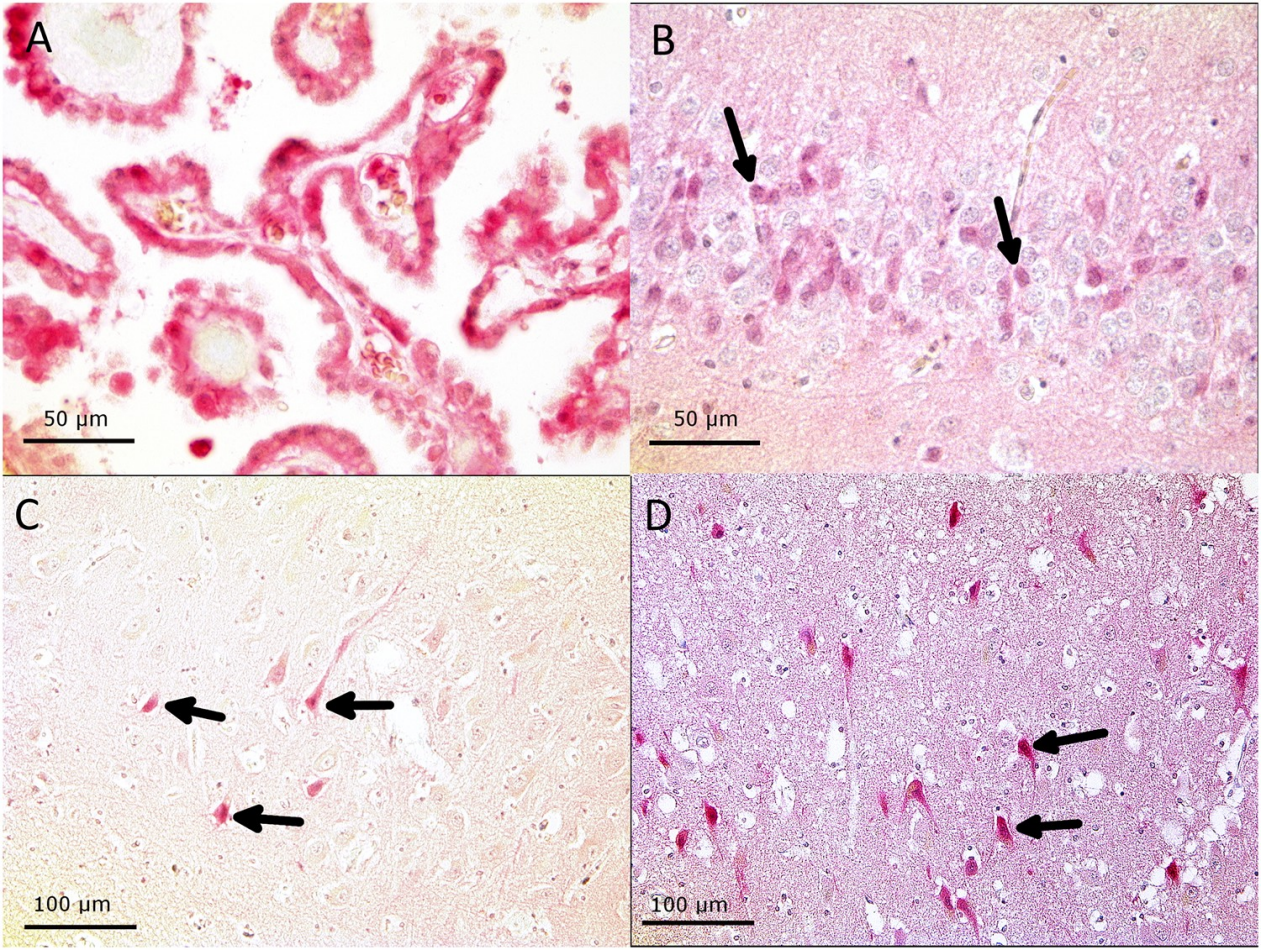
Typical views of fetuin-A immunostaining in the choroid plexus, the dentate gyrus, the cornu ammonis and the cortex. (A) Strong fetuin-A immunostaining (red color) in the choroid plexus, which transports and secretes fetuin-A into the cerebrospinal fluid. (B) Cell-associated fetuin-A immunostaining in the dentate gyrus, (C) the cornu ammonis, and (D) in the cortex.

**Fig 2:**
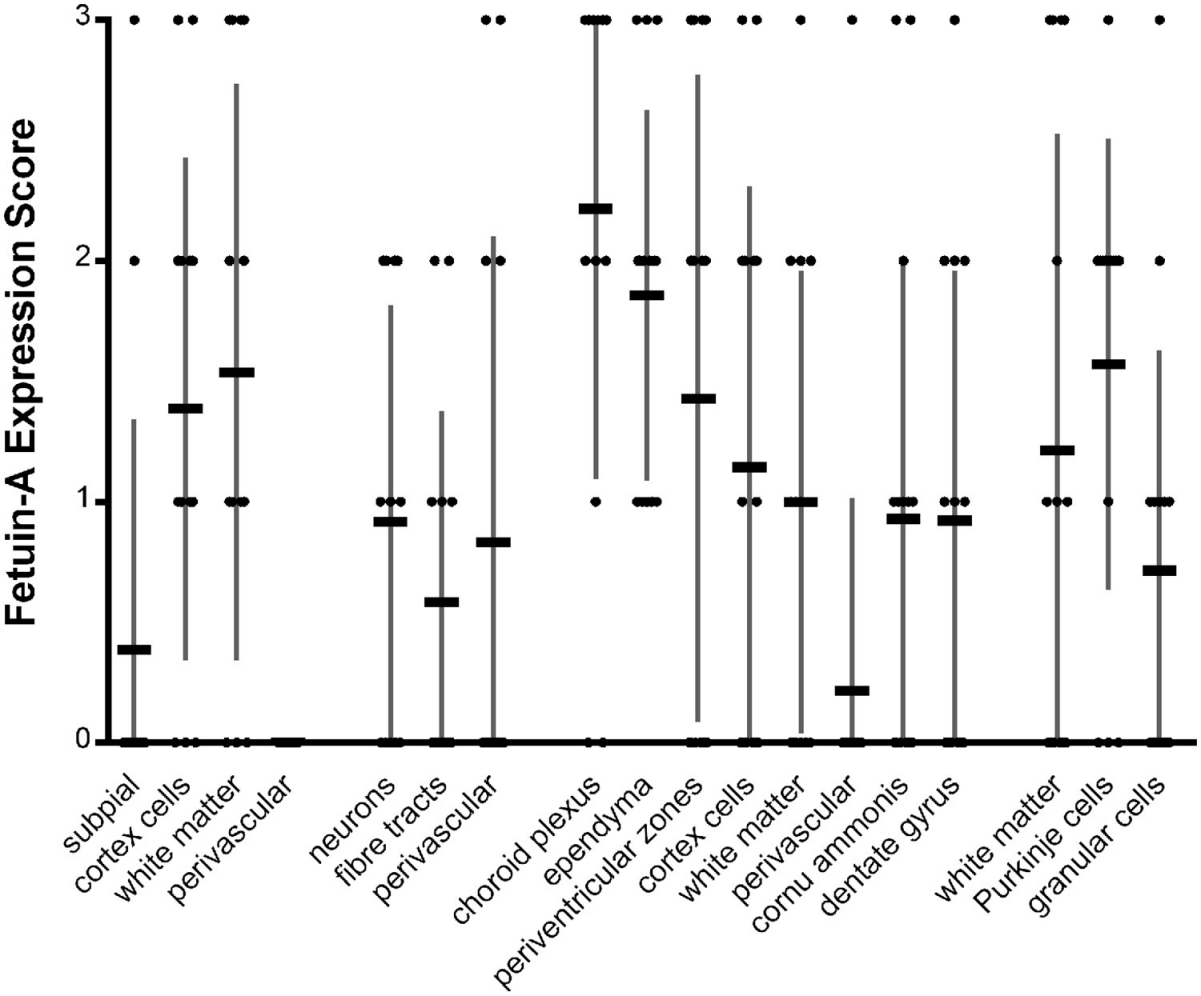
Fetuin-A expression in controls. (Displayed are mean ± SE, individual scores for each case indicated by dots). In the cortex and adjacent white matter fetuin-A-positive cells were equally distributed in cortical and white matter structures. In basal ganglia fetuin-A positive cells were diffusively scattered over all regions. In temporal lobes fetuin-A positive cells were found in choroid plexus, periventricular zones, ependyma, pallium and hippocampus, with highest values in the choroid plexus and ependyma cells. In the cerebellum Purkinje cells were found to strongly express fetuin-A.

First, we studied the distribution of fetuin-A in tissues unaffected by ischemia or inflammation using a semiquantitative score ranging from “0” to “3” (for details see Methods section). We found that fetuin-A was normally present at low levels in all subregions including cortex and adjacent white matter, basal ganglia, temporal lobe and cerebellum. In the cortex and adjacent white matter fetuin-A-positive cells were equally distributed in cortical and white matter structures. In basal ganglia fetuin-A positive cells were diffusively scattered over all regions. In temporal lobes fetuin-A positive cells were found in choroid plexus, ependyma, periventricular zones, cortex, white matter and hippocampus, with highest values in the choroid plexus and ependymal cells. Over all regions, except choroid plexus, mean scores never exceeded a fetuin-A expression score higher than 2. In the cerebellum Purkinje cells were found to strongly express fetuin-A. In negative controls all examined structures, including choroid plexus, stained negative for fetuin-A. Fig 1A–1D show typical views of fetuin-A-positive staining in the choroid plexus, the dentate gyrus, the cornu ammonis and the cortex. Fig 2 shows the mean fetuin-A distribution in controls.

### Focal fetuin-A staining is restricted to the white matter of ischemic brain tissue

Thirty-three cortex/white matter tissue samples from 20 patients with inflammatory diseases and 13 samples from 8 patients with ischemia were available for further analysis. Inflamed tissue showed similar mean fetuin-A staining like controls. In cortical tissues of inflamed brains fetuin-A-positive cells appeared pyramidal, round or star shaped. Fetuin-A staining was slightly elevated in ischemic lesions, yet the elevation did not reach statistical significance.

**Fig 3:**
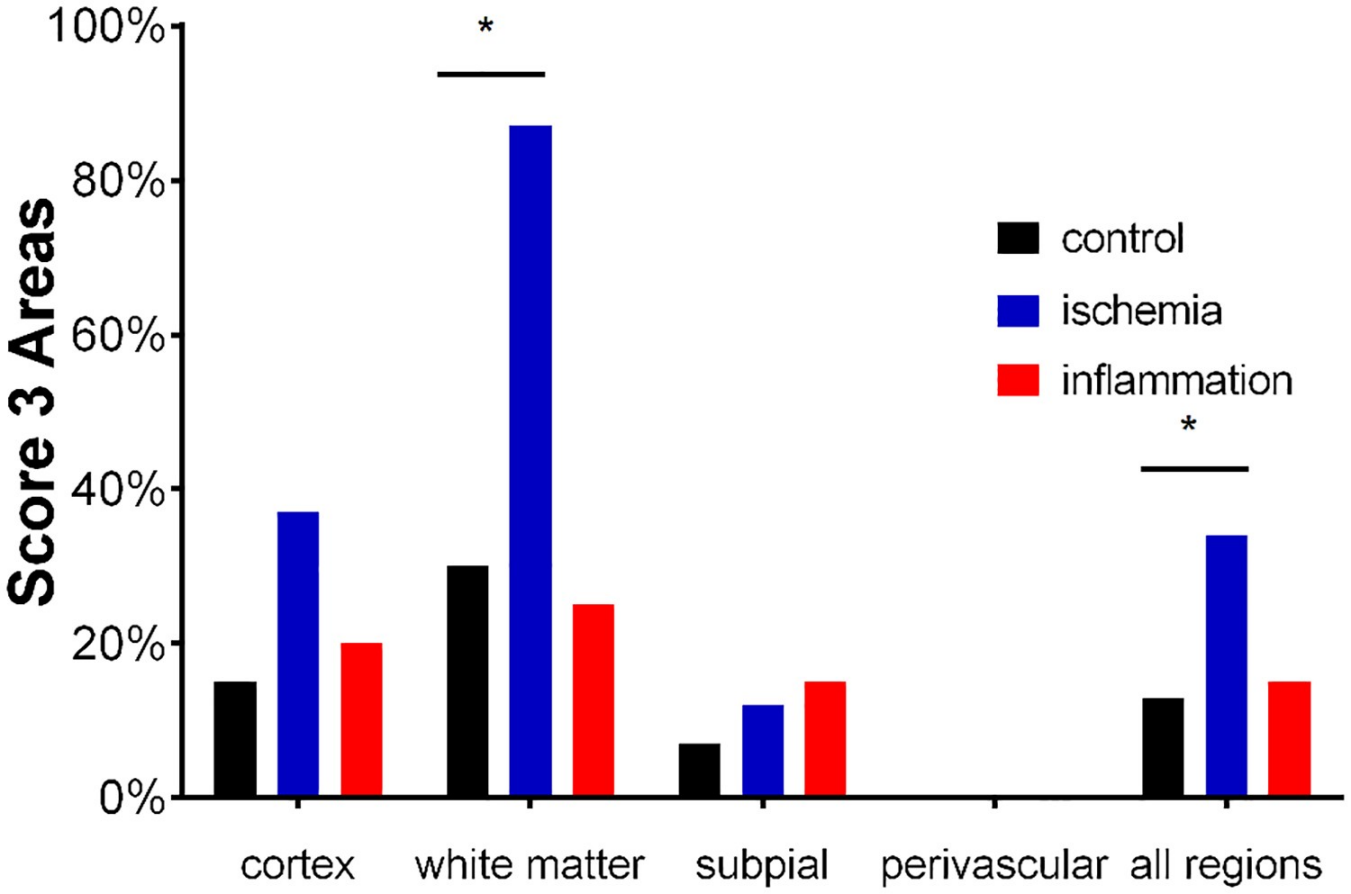
Fetuin-A expression of score 3 areas in cortex and adjacent white matter. Strong focal staining was significantly more frequent in ischemic lesions compared to controls mainly due to focal staining in the white matter of ischemic brains. Inflammatory tissue, in contrast, did not show altered immunoreactivity compared to control brain tissue. Cerebral tissues of 20 inflammatory cases, 8 ischemic cases and 13 control cases were evaluated. * p < 0.05.

The fact that diseased samples did not significantly differ from controls with regard to overall fetuin-A staining intensity was explained by the fact that control sections had background staining for fetuin-A as expected for a highly expressed plasma protein. We asked if differences in staining intensity were present in the strong (score 3) fetuin-A positive staining category. Fig 3 illustrates the percentage of score 3 areas in cortex and adjacent white matter. Strong focal staining was indeed significantly more frequent in ischemic lesions compared to controls (p = 0.030), mainly due to focal staining of cells in the white matter of ischemic tissue (p = 0.024). Inflammatory tissue, in contrast, did not show altered immunoreactivity compared to control brain tissue.

### Basal ganglia of inflammatory and control cases show sparse, scattered fetuin-A positive cells

Among 18 tissue samples from 18 inflammatory cases and 12 tissue samples from 12 control cases, fetuin-A-immunoreactive cells were diffusely scattered over all subregions. With mean staining scores below 1 there were no differences between both groups. Focal accumulations were only detected in 3 tissue samples: in the perivascular region of two controls and along fiber tracts in one inflammatory case.

### Temporal lobe—Strongly positive fetuin-A staining of choroid plexus and ependyma

**Fig 4:**
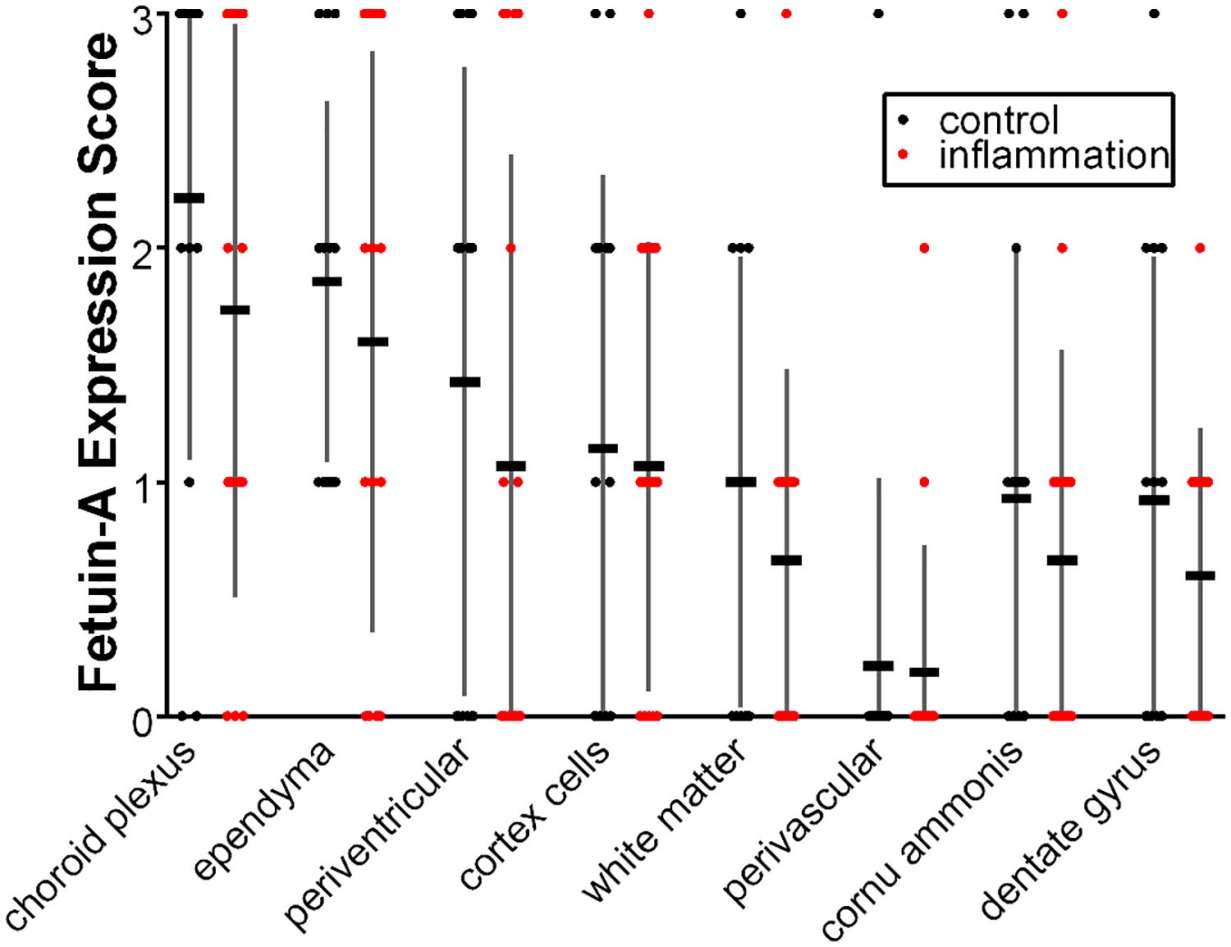
Fetuin-A expression in the temporal lobe of inflammatory cases (n = 16) and controls (n = 14). (Displayed are mean ± SE, individual scores for each case). Compared to controls tissue samples from patients with inflammatory diseases tended to show lower staining scores although the difference was not significant.

As for temporal lobes, 8 subregions were evaluated for the presence of fetuin-A. Fig 4 shows the mean fetuin-A distribution in the temporal lobe of inflammatory cases (n = 16) and controls (n = 14). Compared to controls, tissue samples from patients with inflammatory diseases tended to show lower staining scores although the difference was not significant.

**Fig 5:**
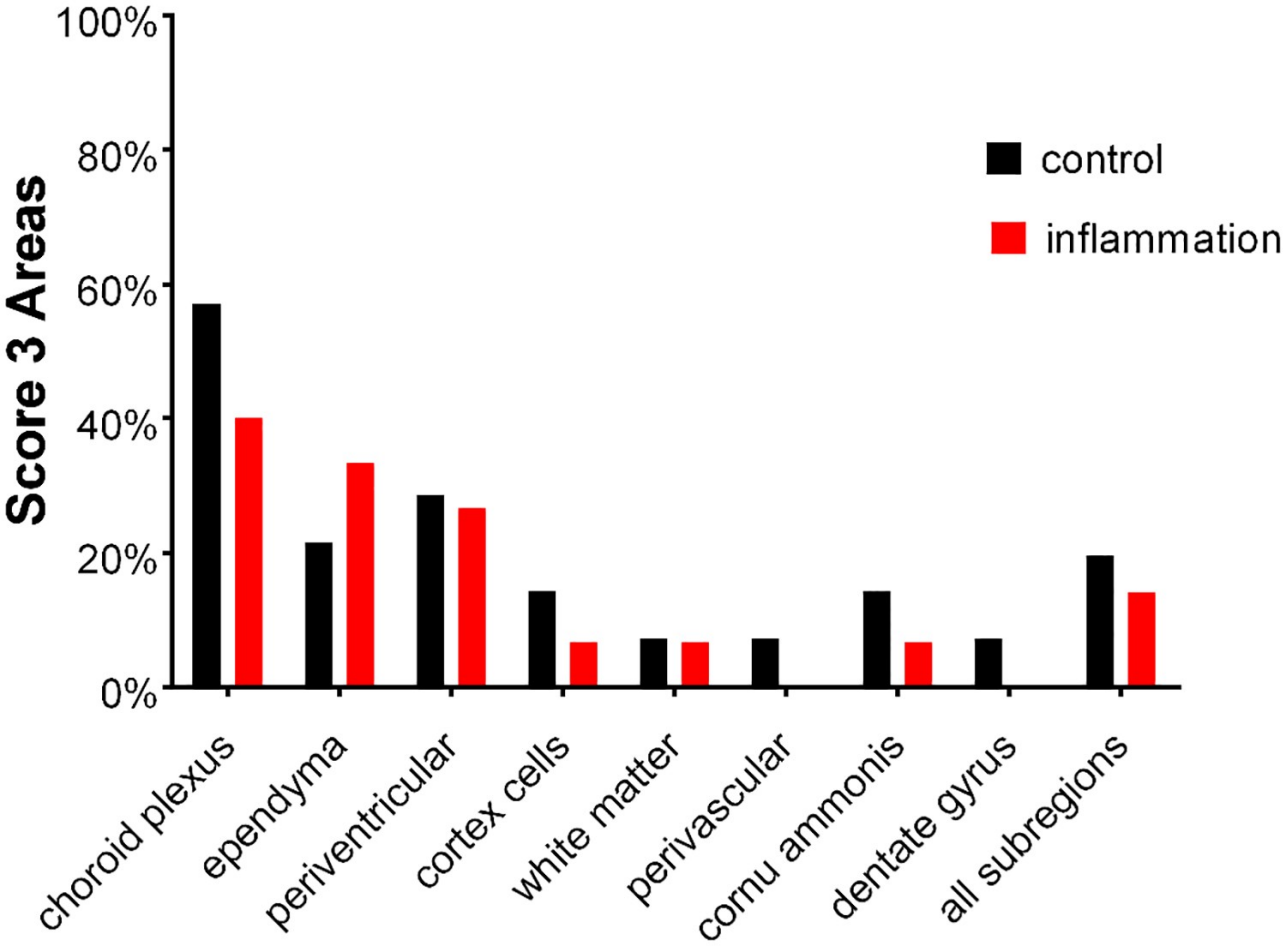
Percentage of score 3 areas in the temporal lobes. In both groups, strongest fetuin-A staining was detected in the choroid plexus cells. Focal accumulations (“score 3” areas) were also frequent in ependyma cells and along the periventricular zones. We further detected focal accumulations of fetuin-A positive cells in temporal cortex, the adjacent white matter and hippocampus of some cases. Within each subregion Fisher´s exact test did not show significant differences between the control and inflammation groups. Between subregions however, there were statistically significant differences in score 3 areas in that e.g. choroid plexus showed 40–57% score 3 staining intensity and white matter showed less than 10% score 3 staining intensity.

Fig 5 illustrates the percentage of score 3 areas in the temporal lobes. In both groups, strongest fetuin-A staining was detected in the choroid plexus cells. Focal accumulations (“score 3” areas) were also frequent in ependyma cells and along the periventricular zones. We further detected focal accumulations of fetuin-A positive cells in temporal cortex, the adjacent white matter and hippocampus of some cases. Within each subregion Fisher´s exact test did not show significant differences between the control and inflammation groups. Between subregions however, there were statistically significant differences in score 3 areas in that e.g. choroid plexus showed 40–57% score 3 staining intensity and white matter showed less than 10% score 3 staining intensity.

### Cerebellar cells in inflammatory cases show reduced fetuin-A staining

**Fig 6:**
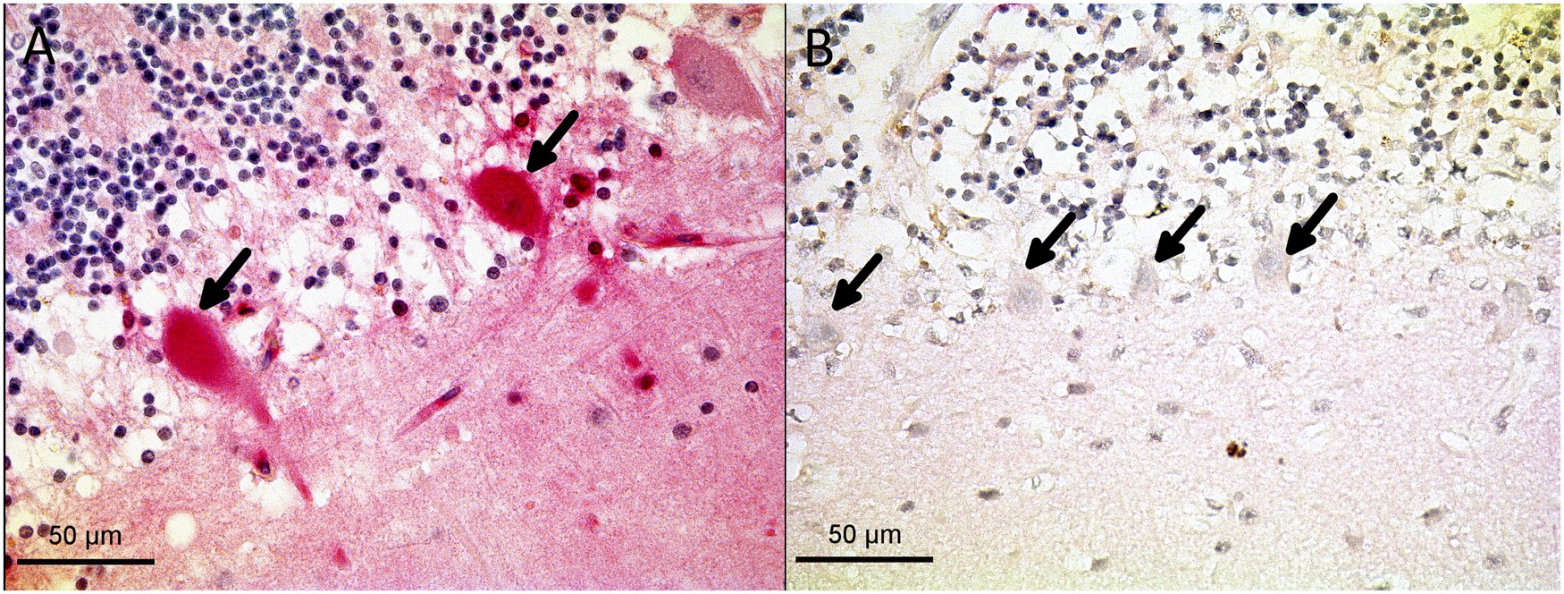
Immunoreactivity of Purkinje cells in inflammatory and control cases. (A) Fetuin-A staining (pink) of Purkinje cells (arrows) in non-inflamed brain tissue. (B) Purkinje cells in brain tissue of a patient with meningitis showed no fetuin-A immunoreactivity.

**Fig 7:**
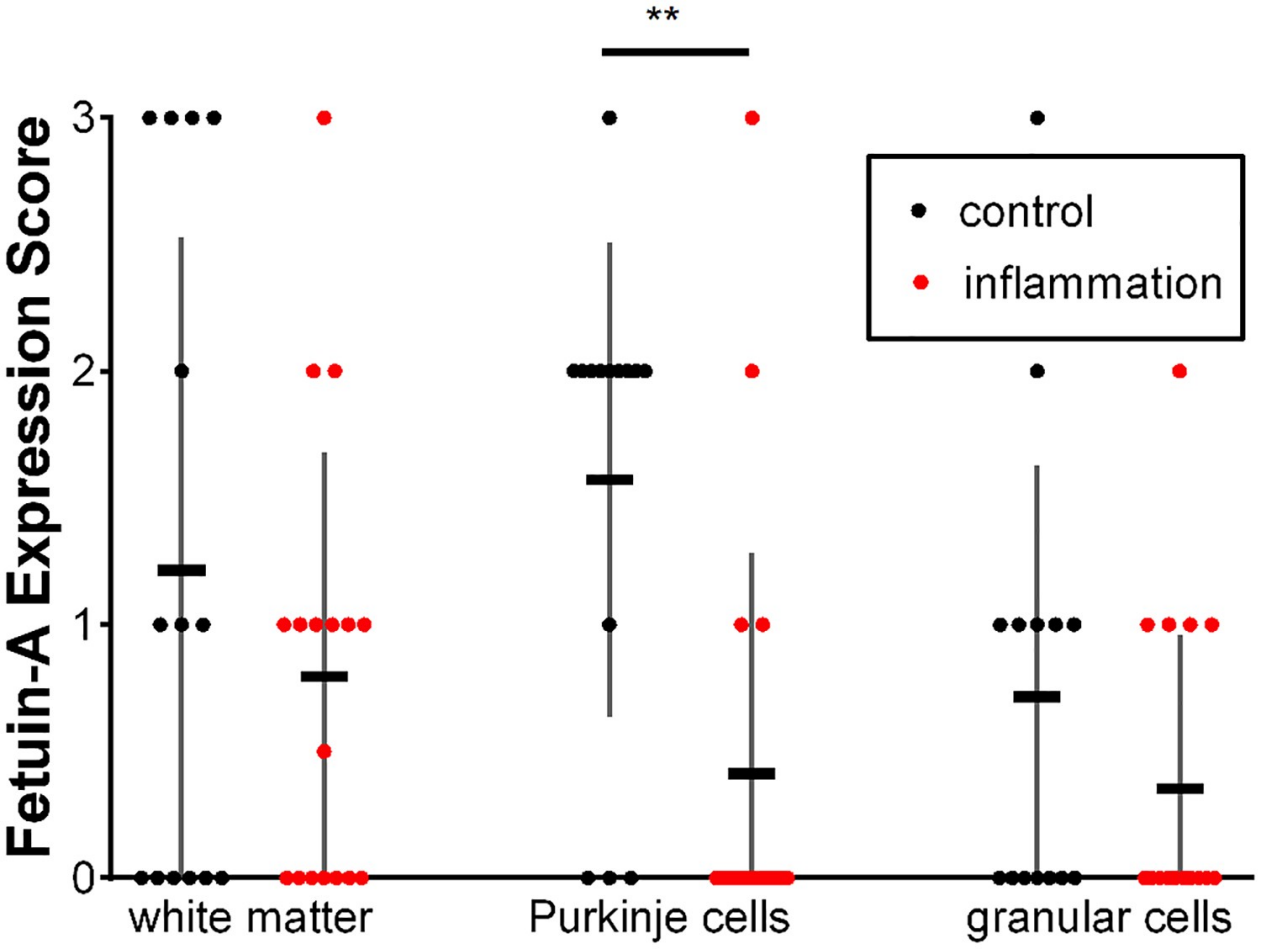
Fetuin-A expression in the cerebellum of inflammatory cases (n = 17) and controls (n = 14). (Displayed are mean ± SE individual scores for each case.) Fetuin-A immunoreactivity was generally decreased in inflammation, reaching statistical significance in the case of Purkinje cells (**p = 0.001). Fetuin-A immunoreactivity also tended to be lower in cerebellar white matter and granular cells, which however, did not reach statistical significance due to large variation.

Cerebellar tissue from 17 different inflammatory cases and 14 control cases was compared. The most striking finding in cerebellar tissue was a significant decrease of fetuin-A expression in Purkinje cells of inflamed tissue: Fetuin-A positive Purkinje cells were detected in 11 out of 14 control cases, but only in 4 of 17 inflamed brains. Fig 6 shows a typical view of Purkinje cells in control and inflammatory cases. Fig 7 illustrates the mean distribution of fetuin-A in the cerebellum of inflammatory cases and controls. Fetuin-A immunoreactivity was generally decreased in inflammation, reaching statistical significance in the case of Purkinje cells (p = 0.001). Fetuin-A immunoreactivity also tended to be lower in cerebellar white matter and granular cells which, however, did not reach statistical significance due to large variation. Focal accumulations of positive cells were most often detected in the cerebellar white matter (4 controls and 1 inflammatory case.

### Ischemic tissue shows heterogeneous fetuin-A-staining

We asked whether fetuin-A distribution changed with lesion progression. To this end, we staged ischemic lesions by HE histology and analysed fetuin-A distribution by immunostaining. Altogether, we analysed 19 tissue samples derived from 14 cases. We detected fetuin-A positive staining of ischemic tissue in eight samples derived from eight cases. Two additional samples from the same cases stained negative for fetuin-A. Eleven tissue samples from six cases stained fetuin-A-negative. In summary, we did not detect stage-dependent differences in fetuin-A immunostaining in that similar fetuin-A positive staining was observed in tissue classified as resolving, organizing and liquefactive necrosis.

### Fetuin-A distribution in pathogen-associated inflammation

To evaluate whether the presence of pathogens led to changes in fetuin-A staining in inflammatory tissue we selected tissue in which pathogens had been identified during routine diagnostics. Fetuin-A-positive staining was observed in 4 of 19 tissue samples from 11 cases, including *Aspergillus species* infection in three, and *human immunodeficiency virus* (HIV) infection in one case, respectively. 15 of 19 tissue samples, in turn, were evaluated as fetuin-A-negative. Pathogens in the latter samples were fungi, including *Aspergillus* (n = 4), *Cryptococcus* (n = 2) and non-specified hypha (n = 2), but also infections with coccus (n = 5) and rod-shaped bacteria (n = 1), and HIV, *Epstein-Barr-Virus* (EBV) and *Cytomegalovirus* (CMV) (n = 1). Thus, no uniform fetuin-A staining pattern in response to infection could be detected.

### Astrocytes but not activated microglia cells are fetuin-A immunoreactive in adult brains

**Fig 8:**
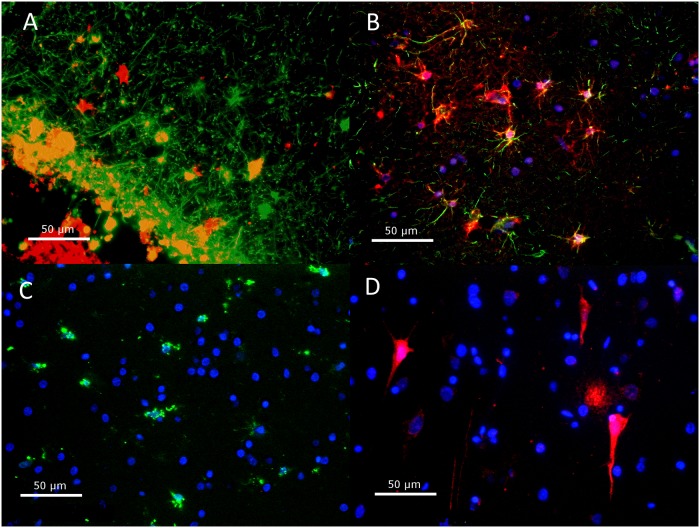
Identification of cells by double immunostaining. Co-localizations of fetuin-A (red) with GFAP (green) in the subpial area of a control case **(A)** and in the cortex of an inflammatory case **(B)**. Activated microglia were detected in every tissue sample, including control cases. The presence of activated microglia was confirmed by positive CD68 staining and the typical amoeboid morphology of microglia **(C)**. Fetuin-A-positive pyramidal neurons could be identified in their characteristic shape and localization **(D)**. Counterstaining with DAPI (blue).

Whereas neocortical and hippocampal neurons could be identified morphologically, specification of smaller cells was more difficult based on morphological criteria alone. Previous studies on neonatal rat brain suggested that fetuin-A is expressed at increased levels in astrocytes and activated microglia [23]. Microglial cells are involved in inflammatory processes in the brain occurring after infection or ischemia. To evaluate whether fetuin-A colocalized with astrocytes or activated microglia in mature brain tissue, we performed double immunostaining of brain tissue sections from all patient groups. The sections were studied for co-localization of fetuin-A with GFAP (astrocytes) and with CD68 (activated microglia, macrophages and monocytes). Fetuin-A co-localized with GFAP, but not with CD68 in all groups. Fig 8 illustrates the identification of cells by immunostaining, showing co-localizations of fetuin-A with GFAP in the subpial area of a control case (Fig 8A) and in the cortex region of an inflammatory case (Fig 8B). Activated microglia were detected in every tissue sample, including control cases. The presence of activated microglia was confirmed by positive CD68 staining and the typical amoeboid morphology of microglia (Fig 8C). Fetuin-A-positive pyramidal neurons could be identified by their characteristic shape and localization (Fig 8D).

To further specify the pattern of GFAP and fetuin-A colocalization in different tissues we studied 25 tissue samples, which previously appeared fetuin-A positive by chromogenic staining: 10 tissue samples of the pallium, 5 tissue samples of basal ganglia, 5 tissue samples of the hippocampus and 5 tissue samples of the cerebellum. Co-localizations of GFAP and fetuin-A were only detected in the pallium and in the hippocampus. In the pallium, GFAP and fetuin-A co-localization was detected in 4 tissue samples from all groups, located in the subpial area and in the grey matter. Among 10 tissue samples of the pallium that were studied, GFAP positive structures were mainly located in subpial and grey matter tissue, but also within white matter. Fetuin-A-positive cells were fewer in number; in the grey matter they showed the characteristic shape of neurons. Fetuin-A positive structures also appeared as small spots within the white matter of the entire tissue sample. In the hippocampus, co-localization of fetuin-A and GFAP was detected in the periventricular zone of an inflamed brain. In tissue samples of 2 control cases and 3 inflammatory cases GFAP-positive cells were frequent in subpial, grey matter, white matter and periventricular zone of the temporal lobe, whereas fetuin-A staining was mostly restricted to the periventricular zone.

To determine whether fetuin-A is also present in activated microglia of inflammatory and ischemic human brain tissue, we examined 21 tissue samples, which previously appeared fetuin-A positive by chromogenic and fluorescence staining: 6 control cases, 8 inflammatory cases and 6 ischemic cases. CD68-positive structures appeared as monocytes inside blood vessels and in cells with the characteristic amoeboid morphology of microglia. Activated microglia were detected in every tissue sample, including control cases. Fetuin-A-positive structures showed a similar distribution as in double staining with GFAP. There was no co-localization of fetuin-A and CD68.

## Discussion

Here we studied the distribution of fetuin-A in mature human brain tissue. Previously, fetuin-A expression had been reported to be absent in the brain of adult sheep [17] [18] [19], rats [20] and neurologically healthy human adults. Unlike these previous studies we detected fetuin-A in all patient groups studied, inflammatory, ischemic as well as control cases, albeit with considerable intragroup and interindividual variation. Thus, in addition to its role in mineralized matrix metabolism, our data indicate that fetuin-A may also be involved in cell and tissue remodeling in the adult brain following inflammatory as well as ischemic brain damage. Here we employed high sensitivity techniques to detect fetuin-A including high amplification anti-alkaline phosphatase (APAAP) and tyramide signal amplification [30] [31]. Previous studies used staining techniques with lower levels of amplification. Despite using high amplification techniques in the majority of the tissue samples studied here, fetuin-A-positive cells were only sparsely or very sparsely scattered in the various tissues; mostly the mean scores did not exceed level 2.

Most intense fetuin-A staining was detected in choroid plexus and ependyma whereas positive staining in the perivascular zone was only detected in a few samples. This finding confirms others´ and our own previous observations on the developing brain of human fetuses and neonatal rats [23]. It supports the theory that plasma-derived hepatic fetuin-A enters the brain at the choroid plexus via cerebrospinal fluid. Little is known about fetuin-A gene regulation in the choroid plexus, an established extrahepatic site of fetuin-A mRNA expression [14].

Fetuin-A staining was also frequently detected in cortex and white matter where it could be attributed to distinct cells, including cortical and hippocampal neurons as well as neurons of the dentate gyrus and cerebellum. In addition to neuronal cells we located fetuin-A within astrocytes by double staining, again in line with our observations on the developing brain [23]. Co-localization of fetuin-A with CD68 was previously reported in the developing brain. Mature brain tissue in this present study showed no co-localization of fetuin-A with CD68-positive cells. This result indicates that fetuin-A may fulfill different functions in inflamed adult compared to the developing brain. Nevertheless, the observation that CD68-positive activated microglial cells were regularly detected in fetuin-A-containing tissue supports the notion that fetuin-A protein accumulates at sites of cellular stress, suggesting that fetuin-A is associated with natural tissue remodeling in the developing brain and with pathological remodeling of diseased brain regions.

In their animal model of cerebral ischemia Wang et al. [8] found an increase of fetuin-A levels in brain tissue of rats after arterial occlusion which started between 2h and 6h, was significant after 24h, peaked at 48h and significantly decreased after 72h. In our study, focal accumulations of fetuin-A-positive cells were significantly more frequent in the white matter in the vicinity ischemic lesions compared to controls. The increase of fetuin-A in ischemic rat brain in the study by Wang et al. [8] started after 2h. Fetuin-A-distribution in ischemic lesions of various stages did not indicate a stage-related pattern. However, the ischemic tissue samples were obtained from areas that had turned necrotic several days before autopsy. Therefore fetuin-A levels might already have been decreased to baseline levels before specimen sampling.

In inflammatory cases we did not find an increase but a significant decrease of fetuin-A staining, which was confined to Purkinje cells of the cerebellar cortex. As the hepatic synthesis of fetuin-A is down-regulated by pro-inflammatory cytokines, a matching down-regulation of fetuin-A protein expression is to be expected in the brain. Purkinje cells were a very striking example of fetuin-A down regulation. Purkinje cells were positive in most of the control cases yet in few inflammatory cases only, although being clearly visible in these inflammatory tissues. The decreased fetuin-A staining in infectious diseases contrasts the situation in multiple sclerosis, where fetuin-A staining of Purkinje cells was increased. [27] Purkinje cells are thought to integrate signals from other neurons of the cerebellar cortex to send inhibitory projections to the cerebellar nuclei, which control motor and vestibular functions and may thus be considered sentinels of the surrounding brain tissue.

We did not determine differences in fetuin-A staining with regard to the cause of infection, e. g between patients with viral, fungal or bacterial infection, respectively. Fetuin-A and CD68 double staining identified CD68-positive microglia in tissues of all groups also staining strongly positive for fetuin-A. This result supports the association of fetuin-A protein accumulation at sites of cellular stress. Unlike in the developing brain [23], but in line with observations of experimental autoimmune encephalitis in mice [27], we could not locate fetuin-A in activated microglia. Whether fetuin-A accumulation is cause or consequence of stress cannot be decided by descriptive immunohistochemistry alone—the fact that fetuin-A treatment of macrophages inhibits HMGB1 secretion and ensuing inflammation [7] strongly suggests that fetuin-A accumulation in cells and tissues is reactive-protective rather than causative of stress. Accordingly, cerebral fetuin-A expression during different inflammatory and infectious conditions should be further studied in experimental animal models.

## Conclusion

We report widespread distribution of fetuin-A protein in the mature human brain where it is already present in physiological conditions. Consistent with previous studies of the animal brain, we observed increased accumulation of fetuin-A during ischemia and decreased staining during inflammation. Experimental studies of the role of fetuin-A in the adult brain, especially during inflammation, are required.

## Supporting information

S1 File Dataset. This file contains the semiquantitative scores for each tissue sample.
(XLSX)
